# Modeling the melting of multicomponent systems: the case of MgSiO_3_ perovskite under lower mantle conditions

**DOI:** 10.1038/srep29830

**Published:** 2016-07-21

**Authors:** Cono Di Paola, John P. Brodholt

**Affiliations:** 1 Department of Earth Sciences, University College London, WC1E 6BT London United Kingdom

## Abstract

Knowledge of the melting properties of materials, especially at extreme pressure conditions, represents a long-standing scientific challenge. For instance, there is currently considerable uncertainty over the melting temperatures of the high-pressure mantle mineral, bridgmanite (MgSiO_3_-perovskite), with current estimates of the melting T at the base of the mantle ranging from 4800 K to 8000 K. The difficulty with experimentally measuring high pressure melting temperatures has motivated the use of *ab initio* methods, however, melting is a complex multi-scale phenomenon and the timescale for melting can be prohibitively long. Here we show that a combination of empirical and *ab*-*initio* molecular dynamics calculations can be used to successfully predict the melting point of multicomponent systems, such as MgSiO_3_ perovskite. We predict the correct low-pressure melting T, and at high-pressure we show that the melting temperature is only 5000 K at 120 GPa, a value lower than nearly all previous estimates. In addition, we believe that this strategy is of general applicability and therefore suitable for any system under physical conditions where simpler models fail.

The melting temperatures of lower mantle minerals are fundamental properties for understanding the evolution of the Earth from an early magma-ocean state to now, and for understanding the likelihood of melting in the current deep Earth (i.e., in Ultra Low Velocity Zones, ULVZs). Orthorhombic MgSiO_3_ perovskite (now bridgmanite^1^) is the end-member of the most voluminous phase in the lower mantle and so its melting temperature is a key cornerstone to any comprehensive understanding of lower mantle melting. Despite this, considerable doubt remains as to its high-pressure melting temperature.

At low pressures (~25 GPa) the melting temperature of bridgmanite is reasonably well constrained from multi-anvil experiments to be between about 2800 and 2900 K (Ito and Katsura)^2^. This agrees well with more recent experiments of Liebske and Frost^3^. At higher pressure, however, there is little agreement between different studies. First of all, there are currently no static experimental measurements on the high-pressure melting T of perovskite above 100 GPa. The early diamond anvil cell experiments of Heinz and Jeanloz^4^ and Knittle and Jeanloz^5^ produced extremely flat melting curves and, therefore, very low high-pressure melting temperatures (<4000 K at 100 GPa). These low melting temperatures have not been reproduced by other experiments or by theoretical estimates, and are likely to underestimate the high pressure melting temperature of bridgmanite. Other than these, the highest pressure static experiments only reach about 60 GPa. The higher pressure melting curve is constrained by a single shock-wave measurement of Akins *et al*.^6^, which produced a melting temperature of 5500 K at 110 GPa.

The lack of high-pressure data has driven many attempts to estimate the melting temperature of bridgmanite by extrapolating the lower pressure data with simple melting equations, or by using theoretical methods such as molecular dynamics. However, these produce widely varying melting curves. For instance, simple extrapolations of the low-pressure data with various different melting equations predict melting temperatures ranging from 6800 K to ~8000 K at 120 GPa (Zerr and Boehler^7^). Lower melting temperatures of around 6000 K at 120 GPa were found using molecular dynamics simulations (Belonoshko^8^) and a temperature near 5200 K was found by Stixrude and Karki^9^. In this study they used the low-pressure melting temperature of Ito and Katsura^2^ to anchor the melting curve and then used the Clapeyron slope from enthalpies and volumes from *ab initio* molecular dynamics (AIMD) simulations to extrapolate the melting curve to higher pressures. An even lower melting temperature of 5000 K at 120 GPa was suggested by Mosenfelder *et al*.^10^ using equations of state constrained from shock-experiments.

It is fair to say, therefore, that current estimates for the melting temperature of the MgSiO_3_ end-member of bridgmanite at 120 GPa range from somewhere between 5000 K and 8000 K.

In this study we estimate the melting temperature of bridgmanite at 25 GPa and 120 GPa using the so-called two-phase model (2P phase) and *ab initio* molecular dynamics. This method avoids overheating issues typical of melting a fully solid system and has been used in a number of studies to produce melting temperatures for a range of materials^11^,^12^,^13^,^14^,^15^,^16^,^17^,^18^,^19^,^20^,^21^,^22^,^23^,^24^,^25^,^26^,^27^. As described in Methods section, a system of coexisting solid and liquid is set up and allowed to evolve through time. If the system melts, we assume it is above its melting temperature, and if it solidifies then it is below the melting temperature. If solid and liquid coexist, we assume it is on the melting temperature. This is an elegant and simple method used many times in the past (Fe, Al^28^,^29^,^30^,^31^,^32^,^33^,^34^,^35^,^36^,^37^,^38^,^39^). However, as discussed below, diffusion is relatively slow in silicate melts even near the melting temperature, and the system takes a long time to melt and appears to coexist at hundreds of degrees above its melting temperature. This is particularly a problem when using *ab initio* forces since the calculations are restricted to 100 ps or so, and we would predict melting temperatures that are too high. We show, however, that we can use the results from much cheaper empirical potential simulations to extrapolate the *ab initio* simulations to much longer timescales and produce very accurate melting temperatures.

## Results

In order to account for the very long times it can take the two-phase system to melt, while at the same time keeping the accuracy of the *ab initio* forces and energies, we characterised the melting kinetics (time to melting) using a classical empirical potential model and extremely long simulation times. To do this we used the Matsui pair-potential^40^. This potential model was designed to describe the MgSiO_3_ perovskite solid crystal over a small range of pressures and temperatures, and produces melting temperatures that are somewhat higher than via first principles (FP) methods. However, as we show later, diffusion rates and coordination numbers are very similar to those obtained *ab initio*, and so we use the classical potential to correct the *ab initio* simulations to infinite time.

To minimise any differences between the classical and DFT results, simulations were run in an NVT ensemble at the *ab initio* volume of the liquid at the target pressure (found via a set of fully liquid NPT simulations at 25 GPa and 120 GPa), using the coexistence approach for the model and recording the time it takes the system to melt as a function of temperature. The detailed procedure can be found in the Supplementary Information.

**Figure 1:**
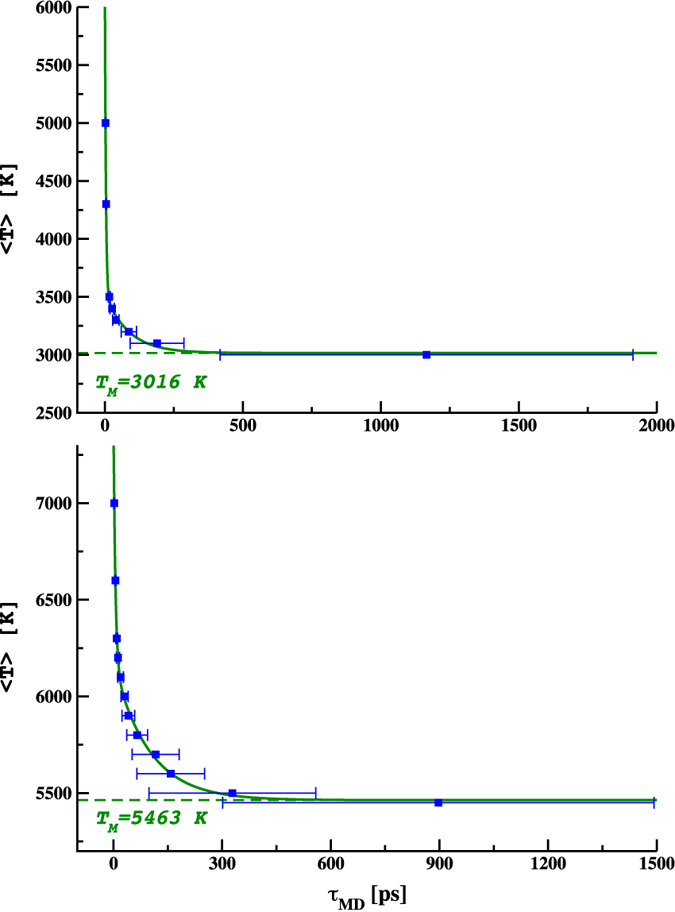
Melting times *τ* (including uncertainties due to the average over 10 trials per temperature) calculated via CMD-NVT ensemble (blue full squares) and curve fitting with 2-phase decay kinetic model 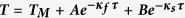. Upper panel: P = 25 GPa, T_*M*_ = 3016 ± 35 K, A = 2729 K, B = 515 K, *κ*_*f*_ = 0.2783 *ps*^−1^, *κ*_*s*_ = 0.01182 *ps*^−1^, %_*f*_ = 84.13% and R^2^ = 0.998. Lower panel: P = 120 GPa, T_*M*_ = 5463 ± 15 K, A = 1242 K, B = 709 K, *κ*_*f*_ = 0.1914 *ps*^−1^, *κ*_*s*_ = 0.01030 *ps*^−1^, %_*f*_ = 63.67% and R^2^ = 0.998. Standard error of the mean at the melting temperature is calculated as 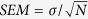, where N = 8 and N = 12 are the number of the interpolated points and *σ* is the standard deviation.

Figure 1 shows the time, *τ*, it took the two-phase system to melt at different temperatures using the classical potential model. The results are shown for two pressures (25 GPa and 120 GPa). At very high temperatures the two-phase system melts very quickly, while *τ* increases dramatically as the temperature of the system approaches the solid-liquid transition point. The error bars are the uncertainties obtained from 10 trials per temperature, and show that the time to melting for the same temperature can vary quite significantly for different starting conditions (i.e., initial atomic velocities). This is particularly pronounced near the melting temperature.

The T-*τ* profile cannot be fit via a single activation energy or rate constant, and suggests that the perovskite melting process is dominated by different kinetics at 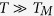 than at temperatures closer to the melting temperature. An explanation for this behaviour comes from an interpretation of the melting process of solids by Samanta *et al*.^41^. Metadynamics calculations showed that the melting of a solid proceeds through multiple barrier-crossing events and competitive pathways when the temperature is relatively close to the melting point. On the other hand, when the system is heated to temperatures much higher than T_*M*_, the solid melts via a single step process with a small activation energy. We have, therefore, adopted a similar multi-barrier formulation to interpolate (*R*^2^ = 0.99) our melting time T-*τ* curve with a ‘2-rate exponential decay’ model:
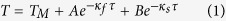
where *T*_*M*_ is the melting temperature at the desired pressure, *κ*_*s*_ and *κ*_*f*_ (in ps^−1^) represent the kinetic constants for the slow and fast processes respectively and the pre-exponential factors *A* and *B* (in Kelvin) define the percentage of the fast process that spans between the fastest point to the melting temperature *T*_*M*_ as follows: 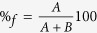.

Using this kinetic model, we tentatively suggest three different behaviours of the two-phase system of MgSiO_3_ around the melting point: 1) close to the melting point the slow kinetics is dominated by very low diffusion coefficients associated to Mg, Si and O in the melt; 2) at higher temperatures faster melting rates are associated to a very low transition barrier due to the solid becoming vibrationally unstable; 3) for temperatures in between the kinetics derives from a balance between the stability of the solid and the diffusivity of the liquid phase. Regardless of the exact mechanisms, this provides a convenient way of parameterising T vs *τ*, and moreover, for obtaining the true melting temperature for a set of simulations well above the actual melting point.

By fitting the two-rate equation we find a melting point of T_*M*_ = 3016 ± 35 K (as standard error of the mean SEM) and T_*M*_ = 5463 ± 15 K at 25 and 120 GPa, respectively. The fit to this is shown in Fig. 1. We also find that the kinetic constants are weakly dependent on the pressure and that the fast rate is one order of magnitude smaller than the slower one. Moreover, it has to be noticed that the statistical value of %_*fast*_ calculated at both pressures (see caption Fig. 1) showed that the fast-kinetics dominated the two-phase to liquid phase transition. As a consequence, the most important points for the fitting were those with *τ* calculated at higher temperatures. When applied to the AIMD data, this could be especially convenient in terms of computational cost, since the cpu time for *ab initio* calculations dramatically increases where the slow kinetics dominates.

**Figure 2:**
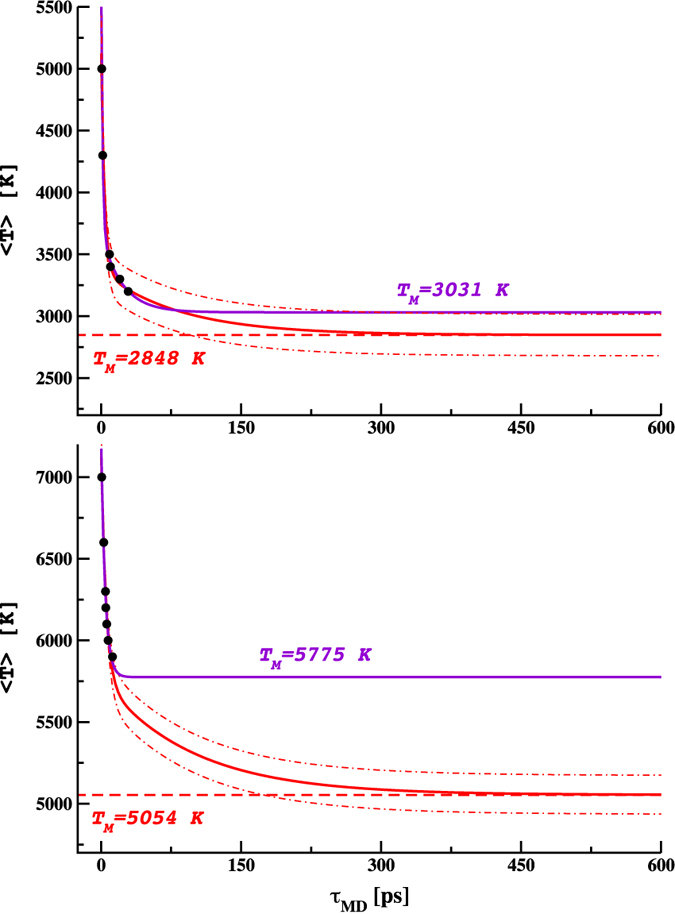
Melting times *τ* calculated via AIMD-NVT ensemble (black full circles) and curve fitting with 2-phase decay kinetic model 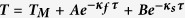. Standard error at the melting temperature is calculated as already explained in Fig. 1 where N = 6 and N = 7. Upper panel: P = 25 GPa, T_*M*_ = 2848 ± 69 K, A = 1809 K and fixed B = 515 K, *κ*_*f*_ = 0.2783 *ps*^−1^ and *κ*_*s*_ = 0.01182 *ps*^−1^, %_*f*_ = 77.84% and R^2^ = 0.978. Unconstrained fitting (violet) curve also included with T_*M*_ = 3031 ± 768 K, A = 2061 K, B = 642 K, *κ*_*f*_ = 0.6564 *ps*^−1^ and *κ*_*s*_ = 0.04536 *ps*^−1^, %_*f*_ = 76.24% and R^2^ = 0.998. Lower panel: P = 120 GPa, T_*M*_ = 5054 ± 45 K, A = 1381 K, fixed B = 709 K, *κ*_*f*_ = 0.1914 *ps*^−1^ and *κ*_*s*_ = 0.01030 *ps*^−1^, %_*f*_ = 66.07% and R^2^ = 0.979. Unconstrained fitting curve for P = 120 GPa has the following form: *T* = *T*_*M*_ + *Ce*^−*κτ*^ and parameters: T_*M*_ = 5775 ± 3327 K, C = 1431 K, *κ* = 0.2272 *ps*^−1^ and R^2^ = 0.987. The red dot-dash lines, defining the area included inside the 95% of statistical confidence (±2*σ*), are only shown for constrained fittings due to the very wide uncertainty found in the unconstrained interpolations.

Figure 2 shows the AIMD results on the same set of two-phase models used with the empirical potentials above. Given the vastly increased computational cost, only the higher temperatures melted within a manageable simulation time (a few 10 s of ps). Adopting the same kinetic model as above, and using the same kinetic constants and prefactor B, this strategy allows us to fit the results with one adjustable parameter, the prefactor A (given in caption of Fig. 2). This results in a melting temperature of T_*M*_ = 2848 K at 25 GPa. At higher pressure the predicted melting point was identified as T_*M*_ = 5054 K, with a standard error below 100 K. To further demonstrate the need for this approach, in Fig. 2 we have also included the fitting curves obtained without the constraints from the CMD simulations. At P = 25 GPa the 2-phase decay model is preserved and a T_*M*_ = 3031 K is obtained which is similar to that when using the rate constants from the classical MD simulations. At P = 120 GPa, however, the model shows 1-phase decay and we obtain a melting temperature of T_*M*_ = 5775 K, ~700 K higher than when using the rate constants from the CMD simulations. Moreover, in both cases the standard error of the melting temperatures is very large. In other words, we would need T vs *τ* results to much lower temperature and much longer simulations to use the *ab initio* calculations alone.

## Discussion

**Figure 3:**
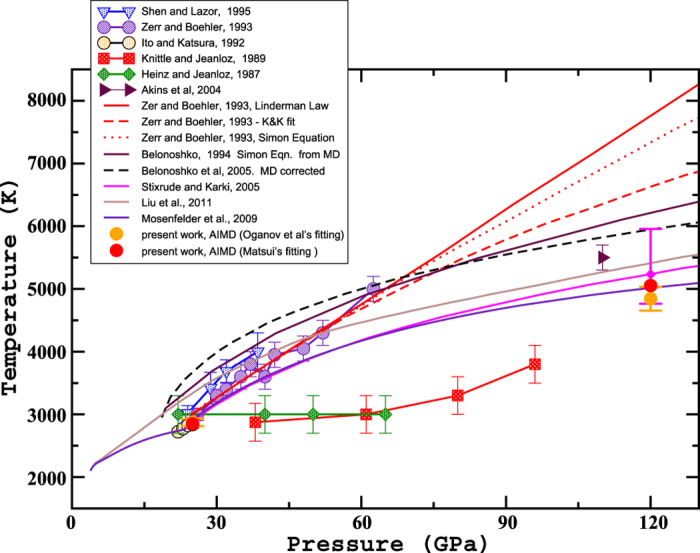
Comparison between present AIMD (full circle) for Matsui’s (red symbols) and Oganov’s (orange symbols) fittings at pressures of 25 GPa and 120 GPa and previous experimental and theoretical results. Present work error bars are smaller than the symbol itself, apart for AIMD point (Oganov’s fitting) at P = 120 GPa. We have also included the value (full magenta diamond symbol) and the error bar (magenta vertical line) around the melting temperature at 120 GPa found by Stixrude *et al*.^9^ for a better comparison with our melting temperature predicted at the same pressure.

The melting temperatures obtained at 25 GPa and 120 GPa are plotted in Fig. 3 together with available experimental data and some other theoretical results. As mentioned above, the classical model tends to overestimate the melting temperature, both with respect to the *ab initio* results and to the low-pressure experimental data of Ito *et al*.^2^. In contrast, the *ab initio* melting temperature at 25 GPa (2848 K) agrees almost perfectly with that obtained experimentally 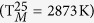 in the multi-anvil cell^2^. At higher pressures we predict a melting temperature 500 K below that obtained from shock-experiments^6^. Our results, also, predict a similar melting temperature (and well within the published uncertainty) to that^9^ extrapolated with a Clapeyron slope based on enthalpies and melting volumes obtained from *ab initio* simulations but fixing the low-pressure results to the value found by Ito *et al*.^2^.

Since we used three kinetic parameters for T vs *τ* obtained from the empirical potentials to obtain the melting temperature of the *ab initio* results, it is worth comparing some properties of the liquid from the two types of simulations. We concentrate on the liquid properties since the Matsui pair-potential^40^ was parameterised for the solid and has already been shown to be a reasonable model for MgSiO_3_ bridgmanite.

**Figure 4:**
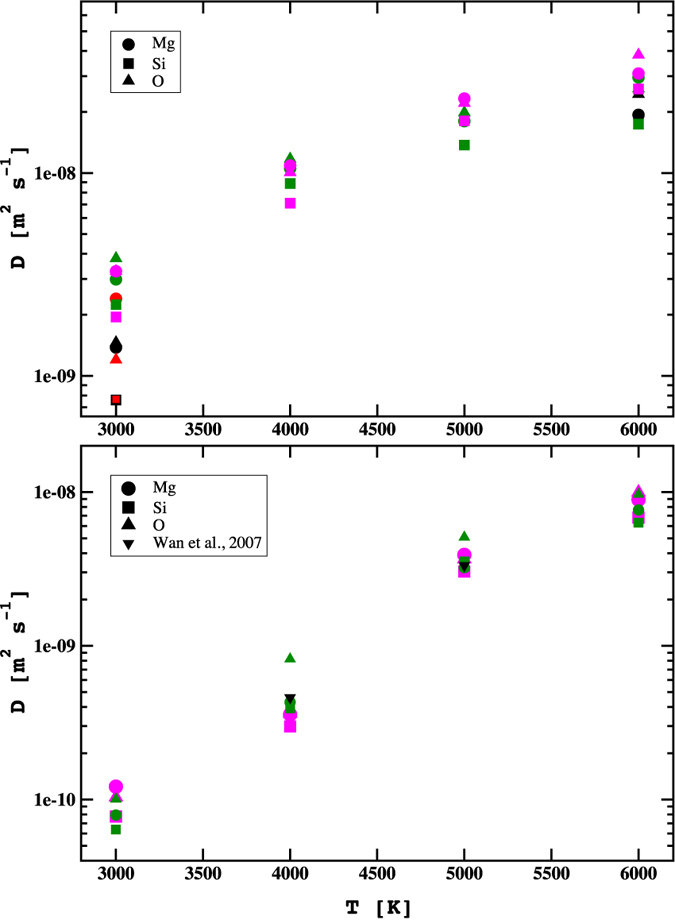
Self diffusion coefficients (DC) of magnesium (full circle), silicon (full square) and oxygen (full triangle up) calculated over mean square displacement at CMD (green) and AIMD (magenta) level. Upper panel: comparison of atomic DC present work at 25 GPa with first-principles simulations^44^ (black symbols) at 25 GPa (3000 K) and 24 GPa (6000 K) and with classical molecular dynamics data (red symbols) at 26.9 GPa^57^ using BKS force field. Lower panel: comparison of atomic DC present work with Car-Parrinello NPT-MD calculations (black full triangle down symbols) at 120 GPa^45^.

Firstly, we suggest above that the kinetics of melting near the melting temperature may be dominated by atomic self diffusivity in the liquid. Diffusion coefficients for the atom types present in the system (Mg, Si and O) were calculated from the mean square displacement (MSD), as described in Allen and Tildesley^42^. The *ab initio* and classical potential results are compared to each other in Fig. 4. As no experimental data are found in the literature, we also compare our classical and *ab initio* values with previous theoretical results. We find a good agreement between our AIMD and CMD data, and with previous classical (see Spera *et al*.^43^), *ab*-*initio*^44^ and NPT Car-Parrinello DFT calculations^45^. We did not observe any dramatic changes in diffusivity between the two approaches either at 25 GPa or at 120 GPa. At higher pressure (Fig. 4, lower panel), our calculations confirmed that the changes of volume and density could be divided into two regions: T < 5000 K, where a slow diffusion (around 10^−9^ m^2^ s^−1^) denoted a very strong amorphisation of the molten bridgmanite reaching very low diffusivity at T = 3000 K (liquid to glass/solid transition); and T ≥ 5000 K, where the diffusivity characterized a fully liquid system.

Secondly, we compare the first-neighbour coordinations of oxygen around silicon atoms between AIMD and the CMD at the same volume. The AIMD results are in agreement with previous works^9^, showing that the coordination number CN_*Si*–*O*_ in the melt varies from 5 at 25 GPa to 6 at 120 GPa with almost no effect due to the temperature. A further increase up to 7 was observed at pressures as high as 500 GPa^46^. Our analysis of the CMD liquid system revealed the same trend found at *ab initio* level, where the CN_*Si*–*O*_ was smoothly increasing with compression, but showed a slightly higher averaged value of CN_*Si*–*O*_ ~ 6.4 at 120 GPa.

Finally, we consider whether the results are sensitive to the particular pair-potential used to constrain the rate parameters. If we use Oganov^47^ instead of Matsui^40^ as the classical potential, we find a very good agreement between the predicted AIMD melting temperatures at P = 25 GPa (T_*M*_ (Matsui’s fitting) = 2848 ± 69 K and T_*M*_ (Oganov’s fitting) = 2872 ± 59 K), despite the fact that the melting temperatures of these two potential models themselves are quite different. At P = 120 GPa the results also agree well and the melting temperatures agree within error (T_*M*_ (Matsui’s fitting) = 5054 ± 45 K and T_*M*_ (Oganov’s fitting) = 4878 ± 190 K). The whole set of data can be found in the Supplementary Information.

In conclusion we studied the melting process of MgSiO_3_ perovskite at pressures typical of the lower mantle by means of empirical potential and first principles molecular dynamics using a 2-phase (solid-liquid) model. Due to the huge *ab initio* computational cost, we provide a way to predict *T*_*M*_ (*AIMD*) using less expensive classical molecular dynamics simulations.

This was done by fitting a T-*τ* curve calculated from the CMD simulations with a ‘2-phase exponential decay’ kinetic model. We then used a similar model on the AIMD melting results obtained far above the melting temperature to obtain the true *ab initio* melting temperature. The low pressure results agree extremely well with the most accurate experimental results. Our predicted melting temperatures are *T*_*M*_ ~ 2850 *K* at 25 GPa and *T*_*M*_ ~ 5050 *K* at 120 GPa.

We believe that the approach presented in this work is of general applicability. It does not depend on the material under study and shows low sensitivity to the potential in use to model the system. Therefore, it may well represent a powerful and computational affordable approach for studying the melting of a wide range of solids under critical conditions, where the classical potentials usually fail.

## Methods

We use both *ab initio* molecular dynamics and classical potential molecular dynamics on a system of 900 atoms (3 × 3 × 5 supercell), starting with a 3:2 ratio between liquid and solid. The two-phase system is prepared from well-equilibrated systems of fully solid and liquid at the required pressure and temperature.

For the AIMD simulations we use the DFT code VASP^48^,^49^ in the Local Density Approximation (LDA) for the exchange-correlation energy^50^. Due to a fairly large dimension of the chosen supercell, the electronic energy is calculated at the Γ point of the Brillouin zone. To reduce the calculation time, we use an energy cutoff of 400 eV and for the finite basis set consequently apply a Pulay correction to the target pressure. In addition a further 2 GPa are subtracted to the pressure for the error associated to the exchange-correlation functional in use^51^,^52^.

For the classical MD we performed the simulations by means of the dl_poly^53^ code, with the MAM0K empirical potential^40^ to approximate the potential energy via Coulombic, van der Waals and Gilbert-type repulsion energy parameters. We also considered a different pair-potential (Oganov *et al*.^47^) to measure the robustness of our computational approach.

In both cases, the simulations use a timestep of 1 fs and a period for the thermostat and barostat of 1 ps with a PMASS of 10^−3^–10^−4^. The canonical ensemble (NVT) is propagated in time using a Nosé-Hoover thermostat^54^, while the CMD pressure (NPT ensemble) is constrained via a Melchionna modification of the general Nosé-Hoover algorithm^55^. Isothermal-isobaric simulations are performed at FP level using a Parrinello-Rahman scheme with implementation of the Nosé-Poincare approach for isothermal sampling^56^.

The 2P system is prepared from well-equilibrated systems of fully solid (FS) and liquid (FL) at the required pressure and temperature. The FL system was melted at very high temperature and then gradually cooled until the desired T and P. This procedure was followed for both classical and *ab initio* MD runs.

## Additional Information

**How to cite this article**: Di Paola, C. and Brodholt, J. P. Modeling the melting of multicomponent systems: the case of MgSiO_3_ perovskite under lower mantle conditions. *Sci. Rep.* **6**, 29830; doi: 10.1038/srep29830 (2016).

## Supplementary Material

Supplementary Information
