# Supplementary material for: Modeling the melting of multicomponent systems: the case of MgSiO3 perovskite under lower mantle conditions
Source: Sci Rep. 2016 Jul 21;6:29830. doi: 10.1038/srep29830 (PMC4956746; doi:10.1038/srep29830)
Supplement: Supplementary Information [file srep29830-s1.pdf]

Modeling the melting of multicomponent systems: the case of $MgSiO_3$ perovskite under lower mantle conditions

Cono Di Paola* and John Brodholt†

Department of Earth Sciences, University College London,

WC1E 6BT London United Kingdom

I. THE COMPLETE METHODOLOGY

A. Building the supercell

To create the 2-phase model of coexisting solid and liquid with the ratio of 2:3, we use a 3x3x2 supercell of solid in the orthorhombic Pbnm (Perovskite) arrangement (360 atoms) and a 3x3x3 fully liquid supercell (540 atoms), both equilibrated at the desired P&T conditions. The 2-phase 900 atom supercell is then constructed from these two as described in Supplementary Fig. 1.

B. Finding the melting temperature

Once a 2-phase cell is formed, it is unlikely to be at thermal equilibrium and so will either start to melt or alternatively crystalize, depending on the conditions. The volume of the two phase simulation is arbitrary, but at a particular temperature it dictates if a system melts or crystalizes. Assuming, for instance, that if V and T are chosen so that the resulting pressure is below the melting curve, it will then start to melt. As it melts, the proportion of melt increases and so the pressure increases too. One of two things then happen; either the system totally melts in which case the final P&T is above the melting temperature, or alternatively, the pressure increases until the solid and liquid are in thermodynamic equilibrium and the proportion of melt and solid left remain the same for the rest of the simulation. In this case the P&T are exactly on the melting curve. This is the standard procedure for the 2-phase melting technique. We chose our volumes to be the volume of the fully molten system at the desired pressure and temperature. In this way, the lowest temperature simulation which melts and the highest temperature simulations which does not, bracket the melting temperature at the desired pressures (25 GPa and 120 GPa). These volumes were found from *ab initio* simulations of the fully melted 900 unit cell at those two pressures for a variety of temperatures.

The same volumes were used for the CMD simulations too. At 120 GPa the pressure obtained from the Matsui[1] potential was 119 GPa, while at 25 GPa, the potential gave a slightly lower pressure of 22 GPa (see also Supplementary Fig. 2 and 3), indicating that the *ab initio* volume would be slightly larger than the CMD volume at the same pressure. We chose, however, to keep the *ab initio* volumes for the CMD melting simulations since we

are interested in using the CMD simulations for the kinetics of melting, and these generally agree better at the same volumes.

The problem for us is that melting starts to become very sluggish as it approaches its melting temperature and you can be fooled into thinking the system is on the melting curve when in fact it is still slowly melting. This is not a problem with the classical simulation which can be run for nanoseconds or more, but it is a problem for the *ab initio* simulations which can be run for perhaps 100 picoseconds at best. This then led us to using the kinetic model described below.

C. Production run in the NVT ensemble

As described above, the volume of the 2-Phase model at different temperatures is adjusted to that found for the liquid. For each chosen temperature T and volume V, we then proceeded as follows:

1. Short supercell equilibration runs of 5ps using the NVT ensemble and a Berendsen thermostat. We did this for 10 starting points using a different set of seeds for the random number generator to obtain the starting atomic velocities. This provided initial systems for ten independent simulations at each temperature to ensure that the final results do not depend on the initial conditions.

2. The equilibrated systems were then used to start the long (production) CMD-NVT (order of nanoseconds) and AIMD-NVT (10s of picoseconds) runs . In the case when the system melted, melting times τ_{MD} were recorded as the elapsed time between the start of the simulation and the time when the system is completely molten. For the CMD-NVT simulations this was done for all 10 different structures, while for the single AIMD-NVT run the starting point was chosen as the 2-Phase structure with the longest life-time during the CMD-NVT simulation (to avoid random melting). Depending on the P-T conditions, some simulations never melted and indeed started to crystalise. This were obviously not used for fitting the kinetic parameters.

D. The kinetic model

In this work we used a 2-phase kinetic model to fit the temperature T with the melting times τ and to predict the *ab initio* melting point at different pressures using data coming from classical MD simulations.

The kinetic model considered here is coming from the 2-phase exponential decay that is normally employed to explain the behaviour of many chemical and biological processes. As already mentioned in the main text, it has the form:

$$T = T_M + Ae^{-k_f \tau} + Be^{-k_s \tau}$$

where T_M represents the temperature for an infinite time simulation (i.e., the actual melting temperature), and k_f and k_s are the kinetic constants for fast and slow process respectively. The exponential pre-factors A and B represent: $A = (T_0 - T_M) * 0.01 * \%_f$ and $B = (T_0 - T_M) * 0.01 * (100 - \%_f)$, where T_0 is the temperature when τ=0 (highest temperature where the solid is unstable) and $\%_f$ is the percentage of the whole process where the kinetics is dominated by the fastest rate.

When we extrapolate the *ab initio* points with the 2-phase model, we allow the A pre-factor to change while B, k_f and k_s from CMD-NVT are fixed. This is to take into account that we only have data with small τ.

II. COMPARISON BETWEEN PAIR-POTENTIALS

We also performed a set of calculations using the Oganov[2] parametrisation of the Buckingham potential in order to see if the results are strongly sensitive to the potential model used in the fitting. The comparison of the data obtained from the two pair-potentials (force-field parameters in Supplementary Tab. I) performed after CMD-NVT ensemble simulations are shown in Supplementary Tab. II and Supplementary Fig. 4, while the *ab initio* points and the predicted melting temperatures can be found in Supplementary Tab. III and Supplementary Fig. 5 respectively. The results using the Oganov et al [2] potential are in very good agreement with those using the Matsui potential, providing support for the method

and results.

---

* Electronic address: c.paola@ucl.ac.uk

† Electronic address: j.brodholt@ucl.ac.uk

[1] Matsui, M. Molecular dynamics study of $MgSiO_3$ perovskite. *Phys. Chem. Miner.* **16**, 234–238 (1988).

[2] Oganov, A. R., Brodholt, J. P. & Price, G. D. Comparative study of quasiharmonic lattice dynamics, molecular dynamics and debye model applied to $MgSiO_3$ perovskite. *Phys. Earth Planet. Inter.* **122**, 277–288 (2000).

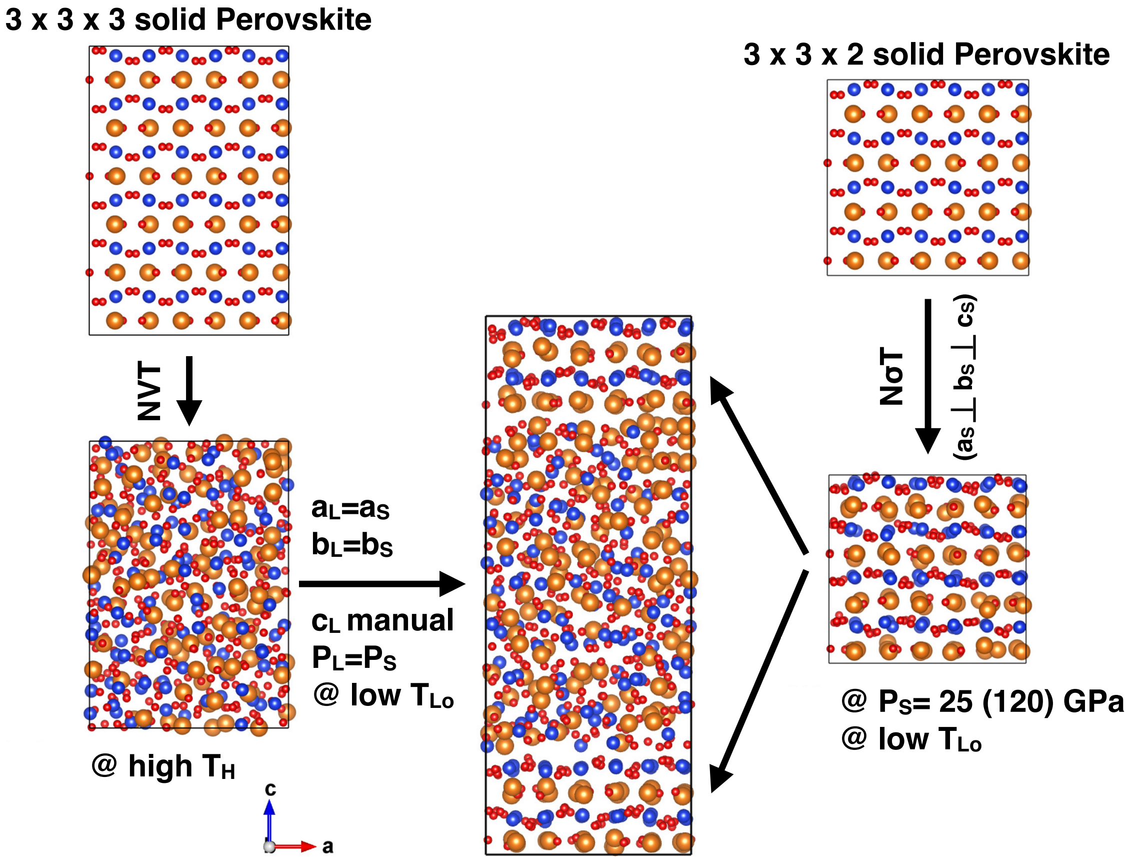

Supplementary Figure 1: Right: constrained CMD-NσT is performed on a solid Perovkite supercell of 3 X 3 X 2 unit cells to obtain the solid part of the 2-Phase at P_S = 25 GPa (120 GPa). In constrained NσT, T is the low temperature $T_{Lo} \geq$ 2500K and the metric tensor is maintained constant to have the same pressure along all the lattice parameters $\mathbf{a}_S$, $\mathbf{b}_S$ and $\mathbf{c}_S$. The lattice parameters are constrained to be orthogonal. Left: the liquid part is obtained performing CMD-NVT on a 3 X 3 X 3 solid Perovskite, where T is a very high temperature ($T_H \geq$ 6000K). Next, the volume is fixed to V=($\mathbf{a}_S$ X $\mathbf{b}_S$ X $\mathbf{c}$) where $\mathbf{c}$ was manually adjusted to have the pressure P_L=P_S (checked with CMD-NVT at T=T_{Lo}). The liquid and solid part are then combined at T= T_{Lo} with a very small gap ($\leq$ 0.5 Å) at the interface to have a final pressure of the 2-Phase supercell P_{2P}=P_S=P_L.

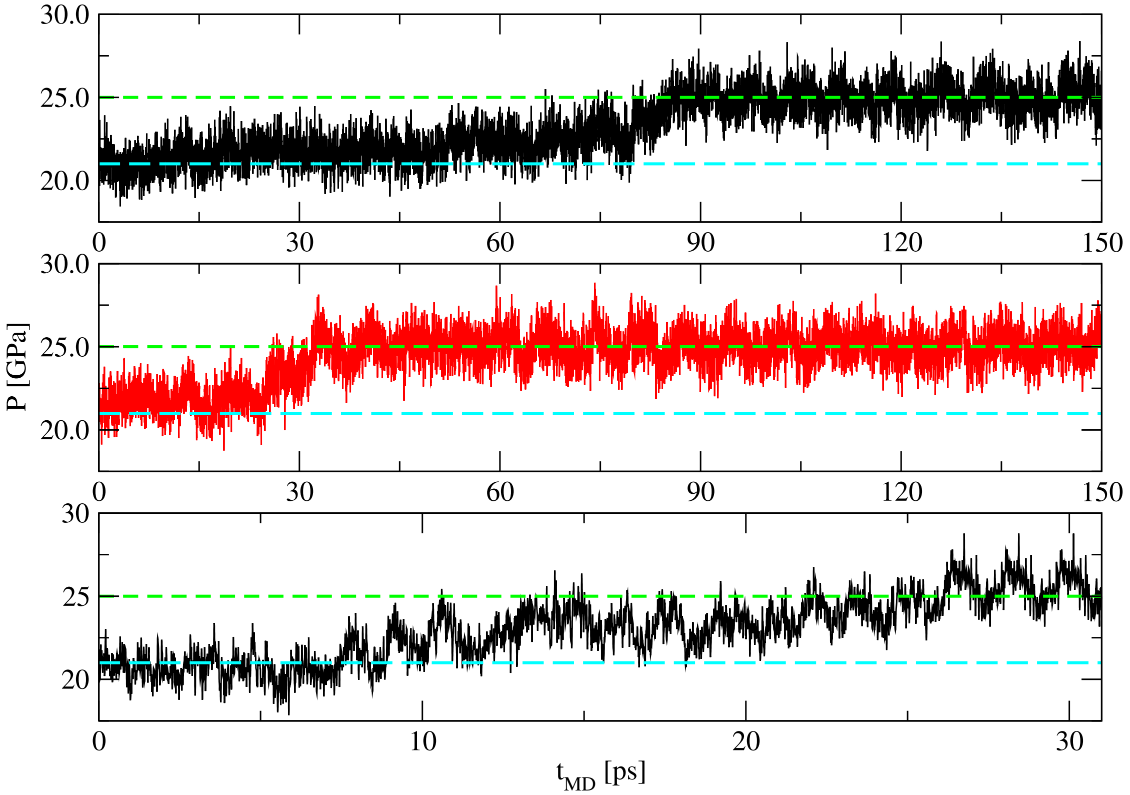

Supplementary Figure 2: Time variation of pressure (in molecular dynamics time t_{MD}) during NVT ensemble simulations at P=25.0 GPa. The green and cyano dashed lines represent the pressure of the completely molten 900-atom model (P_L) and in 2-phase configuration (P_{2P}). Upper and middle panels: 2 different runs (over 10 done) at T=3200K simulated in the CMD-NVT ensemble and with P_L=22.0 GPa and P_{2P}=18.0 GPa, both scaled to match P_L=P_t=25.0. Lower panel: simulation at T=3200K in the AIMD-NVT ensemble and P_L=25.0 GPa (P_{2P}=21.0 GPa).

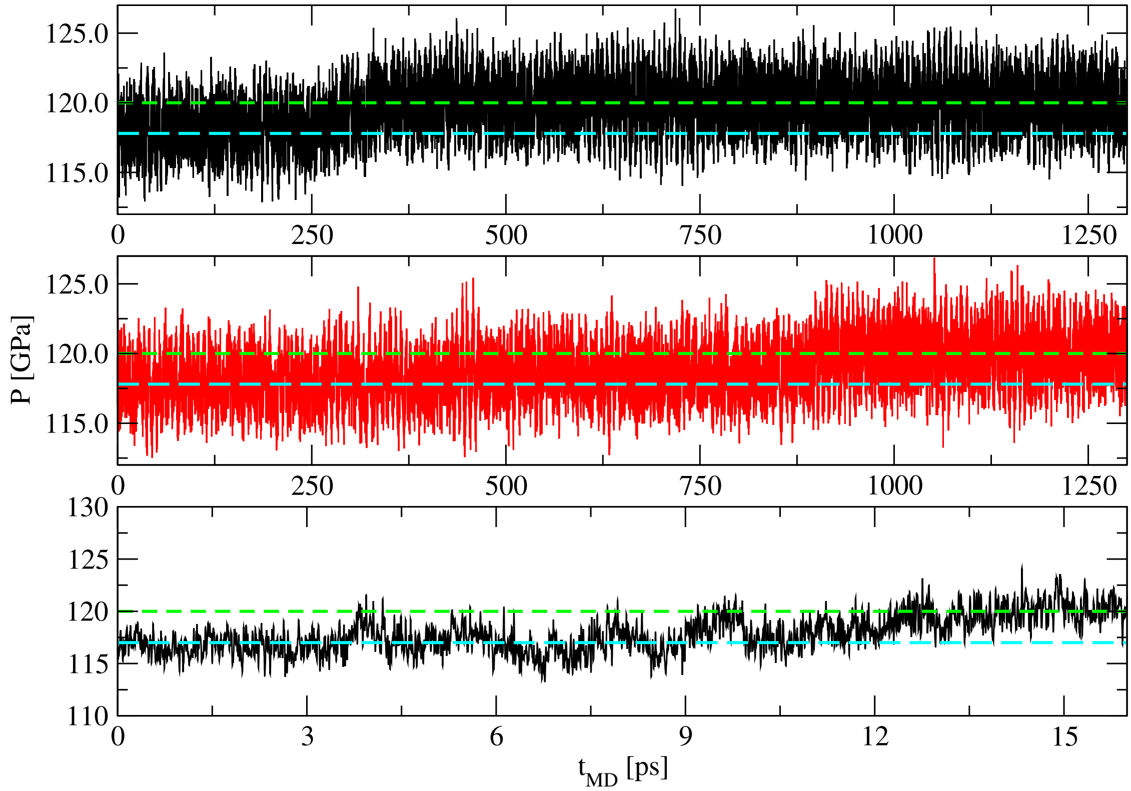

Supplementary Figure 3: Time variation of pressure (in molecular dynamics time t_{MD}) during NVT ensemble simulation at P=120.0 GPa. The green and cyano dashed lines represent the pressure of the completely molten 900-atom model (P_L) and in 2-Phase configuration (P_{2P}). Upper and middle panels: 2 different runs (over 10 done) at T=5500K simulated in the CMD-NVT ensemble and with P_L=119.0 GPa and P_{2P}=116.8 GPa, both scaled to match P_L=P_t=120.0 GPa (P_{2P}=117.2 GPa). Lower panel: simulation at T=5900K in the AIMD-NVT ensemble and P_L=120.0 GPa (P_{2P}=117.8 GPa).

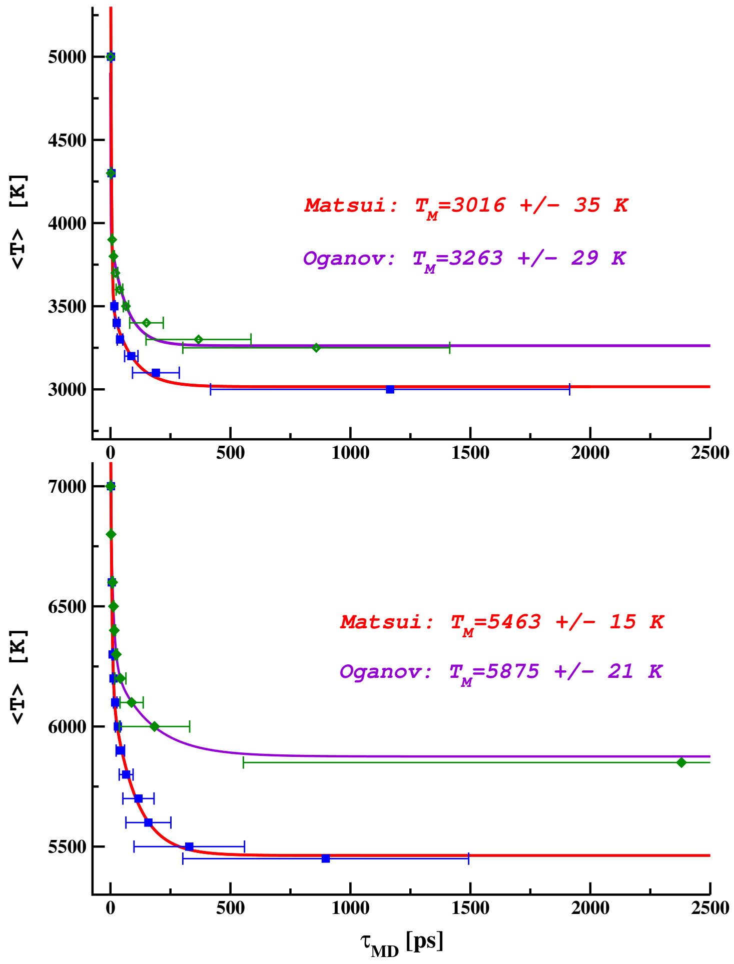

Supplementary Figure 4: Comparison between melting times τ (including uncertainties due to the average over 10 trials per temperature) calculated via CMD-NVT ensemble and curve fitting with 2-phase decay kinetic model $T = T_M + Ae^{-\kappa_f \tau} + Be^{-\kappa_s \tau}$. Upper panel: P_t=25 GPa, Matsui pair-potential (blue full squares and green full diamond): T_M=3016±35 K, A=2729 K , B = 515 K, $\kappa_f = 0.2783 ps^{-1}$, $\kappa_s = 0.01182 ps^{-1}$, $\%_f$=84.13% and R^2=0.998. Oganov pair-potential (green full diamond): T_M=3263±29 K, A=1083 K , B = 654 K, $\kappa_f = 0.9163 ps^{-1}$, $\kappa_s = 0.01531 ps^{-1}$, $\%_f$=62.33% and R^2=0.995. Lower panel: P=120 GPa, Matsui potential: T_M = 5463±15 K, A=1242 K , B = 709 K, $\kappa_f = 0.1914 ps^{-1}$, $\kappa_s = 0.01030 ps^{-1}$, $\%_f$= 63.67% and R^2=0.998. Oganov potential: T_M = 5875±21 K, A=715 K , B = 400 K, $\kappa_f = 0.08847 ps^{-1}$, $\kappa_s = 0.00643 ps^{-1}$, $\%_f$= 64.16% and R^2=0.996. Standard error at the melting temperature is calculated as $SE = \sigma/\sqrt{N}$, where N=8 and N=12 (Matsui), N=9 and N=10 (Oganov) are the number of the interpolated points and σ is the standard deviation.

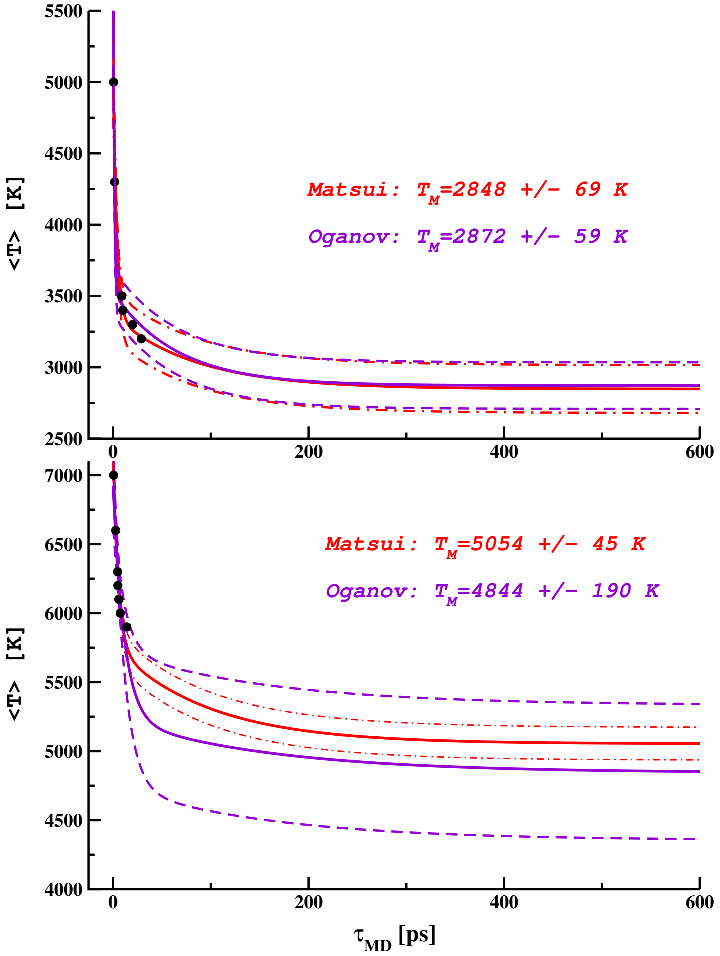

Supplementary Figure 5: Melting times τ calculated via AIMD-NVT ensemble (black full circles) and curve fitting with 2-phase decay kinetic model $T = T_M + Ae^{-\kappa_f \tau} + Be^{-\kappa_s \tau}$. Standard error at the melting temperature is calculated as already explained in Supplementary Figure 4 where N=6 and N=7. Upper panel: P_t=25 GPa, Matsui potential: T_M=2848±69 K, A=1809 K and fixed B = 515 K, $\kappa_f = 0.2783 ps^{-1}$ and $\kappa_s = 0.01182 ps^{-1}$, $\%_f$=77.84% and R^2=0.978. Oganov potential: T_M=2872±59 K, A=2822 K and fixed B = 652 K, $\kappa_f = 0.9163 ps^{-1}$ and $\kappa_s = 0.01531 ps^{-1}$, $\%_f$=81.19% and R^2=0.977. Lower panel: P=120 GPa, Matsui potential: T_M = 5054±45 K, A = 1381 K, fixed B = 709 K, $\kappa_f = 0.1914 ps^{-1}$ and $\kappa_s = 0.01030 ps^{-1}$, $\%_f$= 66.07% and R^2=0.979. Oganov potential: T_M = 4844±190 K, A = 1693 K, fixed B = 400 K, $\kappa_f = 0.08847 ps^{-1}$ and $\kappa_s = 0.00643 ps^{-1}$, $\%_f$= 80.89% and R^2=0.876. The violet dashed and red dot-dashed lines define the area included inside the 95% of statistical confidence ($\pm 2\sigma$).

Supplementary Table I: The Interatomic pair-potential used in this work has the form of a long-range Coulomb interaction and a short-range Buckingham potential which includes the Born-Mayer potential and a van der Waals term, in the form: $V_{ij} = \frac{1}{4\pi\epsilon_0}\frac{q_i q_j}{r_{ij}} + A_{ij} exp\left(-\frac{r_{ij}}{\rho_{ij}}\right) - \frac{C_{ij}}{r_{ij}^6}$

Parameters	Matsui[1]	Oganov[2]
$q_{Mg}/\|e\|$	+1.565	+1.911
$q_{Si}/\|e\|$	+2.329	+2.904
$q_O/\|e\|$	-1.298	-1.605
A_{Mg-O}/eV	8035.120	1041.435
A_{Si-O}/eV	7363.450	1137.028
A_{O-O}/eV	1621.680	2023.800
A_{Mg-Mg}/eV	1309336.700	-
A_{Si-Si}/eV	5005903.500	-
A_{Mg-Si}/eV	2325826.600	-
ρ_{Mg-O}/Å	0.202	0.2866
ρ_{Si-O}/Å	0.190	0.2827
ρ_{O-O}/Å	0.300	0.2674
ρ_{Mg-Mg}/Å	0.104	-
ρ_{Si-Si}/Å	0.080	-
ρ_{Mg-Si}/Å	0.092	-
C_{O-O}/(eVÅ^6)	30.222	13.830

Supplementary Table II: Temperatures T, melting times of the 2-Phase supercell τ_{MD} for CMD-NVT simulations and their standard deviations σ calculated over 10 trials per temperature using Matsui (second column) and Oganov (third column) potentials.

Temperature [K]	$\tau_{MD} \pm \sigma$ [ps] [1]	$\tau_{MD} \pm \sigma$ [ps] [2]
P_t = 25.0 GPa		
5000	2.2 ± 0.8	0.0 ± 0.0
4300	4.4 ± 1.0	1.1 ± 0.8
3900	-	7.2 ± 1.8
3800	-	12.6 ± 3.4
3700	-	20.6 ± 9.7
3600	-	37.5 ± 13.8
3500	16.1 ± 3.4	64.4 ± 11.0
3400	25.8 ± 8.1	153.0 ± 70.2
3300	39.7 ± 11.5	367.3 ± 218.4
3250	-	857.8 ± 556.4
3200	86.7 ± 27.8	-
3100	189.3 ± 97.5	-
3000	1165.6 ± 784.4	-
P_t = 120.0 GPa		
7000	2.0 ± 1.3	0.3 ± 0.9
6800	-	2.4 ± 1.3
6600	5.4 ± 1.6	8.7± 3.0
6500	-	12.5 ± 1.9
6400	-	15.8 ± 5.2
6300	8.9 ± 2.7	24.1 ± 5.1
6200	12.6 ± 4.4	41.3 ± 23.0
6100	19.6 ± 7.4	88.1 ± 48.2
6000	30.6 ± 9.9	183.5 ± 146.6
5900	41.2 ± 17.5	1954.3 ± 1396.7
5850	-	2380.0 ± 1826.2
5800	65.3 ± 28.8	-
5700	116.6 ± 64.9	-
5600	158.0 ± 93.7	-
5500	328.3 ± 230.3	-
5450	897.1 ± 595.7	-

Supplementary Table III: Temperatures T and the 2-Phase supercell melting times τ_{MD} for AIMD-NVT simulations. Note that at 25 GPa the equilibration time for the system is 5ps while at 120.0 GPa it is 10 ps. This time is not included in the melting times, because part of the pre-run and before the production run.

AIMD-NVT	
Temperature [K]	τ_{MD} [ps]
P_t = 25.0 GPa	
5000	0.7
4300	1.7
3500	9.0
3400	10.0
3300	20.0
3200	29.0
P_t = 120.0 GPa	
7000	0.6
6600	2.8
6300	4.7
6200	5.0
6100	6.0
6000	7.5
5900	12.2
